# Taming the SARS-CoV-2-mediated proinflammatory response with BromAc^®^

**DOI:** 10.3389/fimmu.2023.1308477

**Published:** 2023-12-13

**Authors:** Geovane Marques Ferreira, Felipe Alves Clarindo, Ágata Lopes Ribeiro, Letícia Gomes-de-Pontes, Luciana Debortoli de Carvalho, Olindo Assis Martins-Filho, Flávio Guimarães da Fonseca, Mauro Martins Teixeira, Adriano de Paula Sabino, Mathew Suji Eapen, David L. Morris, Sarah J. Valle, Jordana Grazziela Alves Coelho-dos-Reis

**Affiliations:** ^1^ Laboratório de Virologia Básica e Aplicada (LVBA), Departamento de Microbiologia, Instituto de Ciências Biológicas, Universidade Federal de Minas Gerais, Belo Horizonte, Brazil; ^2^ Departamento de Biologia e Biotecnologia de Microrganismos, Universidade Estadual de Santa Cruz (UESC), Ilhéus, Brazil; ^3^ Grupo Integrado de Pesquisas em Biomarcadores, Rene Rachou Institute, Oswaldo Cruz Foundation, Belo Horizonte, Brazil; ^4^ Centro de Tecnologia em Vacinas (CT-Vacinas), Parque Tecnológico de Belo Horizonte, Belo Horizonte, Brazil; ^5^ CT Terapias Avançadas e Inovadoras (CT-Terapias), Universidade Federal de Minas Gerais, Belo Horizonte, Brazil; ^6^ Laboratório de Hematologia Clínica, Experimental e Molecular, Departamento de Análises Clínicas e Toxicológicas, Faculdade de Farmácia, Universidade Federal de Minas Gerais, Belo Horizonte, Brazil; ^7^ Research & Development Department, Mucpharm Pty Ltd, Sydney, NSW, Australia; ^8^ St George and Sutherland Hospital Clinical School, University of New South Wales, Sydney, NSW, Australia; ^9^ Department of Surgery, St George Hospital, Sydney, NSW, Australia; ^10^ Intensive Care Unit, St George Hospital, Sydney, NSW, Australia

**Keywords:** COVID-19, anti-inflammatory, cytokine storm, immunomodulatory, therapeutic strategy, BromAc

## Abstract

**Introduction:**

In the present study, the impact of BromAc®, a specific combination of bromelain and acetylcysteine, on the SARS-CoV-2-specific inflammatory response was evaluated.

**Methods:**

An in vitro stimulation system was standardized using blood samples from 9 healthy donors, luminex assays and flow cytometry were performed.

**Results and discussion:**

BromAc® demonstrated robust anti-inflammatory activity in human peripheral blood cells upon SARS-CoV-2 viral stimuli, reducing the cytokine storm, composed of chemokines, growth factors, and proinflammatory and regulatory cytokines produced after short-term in vitro culture with the inactivated virus (iSARS-CoV-2). A combined reduction in vascular endothelial growth factor (VEGF) induced by SARS-CoV-2, in addition to steady-state levels of platelet recruitment-associated growth factor-PDGFbb, was observed, indicating that BromAc® may be important to reduce thromboembolism in COVID-19. The immunophenotypic analysis of the impact of BromAc® on leukocytes upon viral stimuli showed that BromAc® was able to downmodulate the populations of CD16+ neutrophils and CD14+ monocytes observed after stimulation with iSARS-CoV-2. Conversely, BromAc® treatment increased steady-state HLA-DR expression in CD14+ monocytes and preserved this activation marker in this subset upon iSARS-CoV-2 stimuli, indicating improved monocyte activation upon BromAc® treatment. Additionally, BromAc® downmodulated the iSARS-CoV-2-induced production of TNF-a by the CD19+ B-cells. System biology approaches, utilizing comprehensive correlation matrices and networks, showed distinct patterns of connectivity in groups treated with BromAc®, suggesting loss of connections promoted by the compound and by iSARS-CoV-2 stimuli. Negative correlations amongst proinflammatory axis and other soluble and cellular factors were observed in the iSARS-CoV-2 group treated with BromAc® as compared to the untreated group, demonstrating that BromAc® disengages proinflammatory responses and their interactions with other soluble factors and the axis orchestrated by SARS-CoV-2.

**Conclusion:**

These results give new insights into the mechanisms for the robust anti-inflammatory effect of BromAc® in the steady state and SARS-CoV-2-specific immune leukocyte responses, indicating its potential as a therapeutic strategy for COVID-19.

## 1 Introduction

SARS-CoV-2 is a highly transmissible and pathogenic β-coronavirus that can mutate and evade humoral immune responses induced by previous disease or vaccination. New SARS-CoV-2 variants of concern remain an eminent public health threat, including the highly disseminated omicron variant and its emerging subvariants, such as E.G5 (1–3). The virus is responsible for the coronavirus disease 2019 (COVID-19) syndrome, which may lead to death in immunocompromised and susceptible patients. In addition, COVID-19 is now associated with post-acute sequelae even after convalescence of mild cases, representing one of the biggest challenges to modern civilization, with its rapid spread yet to be eradicated (4).

There are many pathological changes during COVID-19, and these alterations evolve over time. As the course of the COVID-19 disease advances, the proinflammatory responses harness chaos within the host, which is aggravated by hypoxemia and severe and critical illness. Early disease is characterized by exudative neutrophilic capillarity with thrombosis. Late changes that may occur on average after the 10^th^ day of infection include diffuse alveolar damage, intravascular thrombosis, infection, disseminated intravascular coagulopathy (DIC), and proliferation of posterior intra-alveolar fibroblasts (5).

While most SARS-CoV-2-infected individuals present the mild form of COVID-19, some patients experience persistence of symptoms and severe and possibly fatal systemic inflammation and tissue disruption, accompanied by aberrant cytokine storm and neutrophil and monocyte/macrophage recruitment and activation (6–8). The innate immunity acts as the first line of defense against SARS-CoV-2, sensing the virus and activating inflammatory pathways that may or may not promote viral clearance. These innate immune processes associated with SARS-CoV-2 viral recognition and the inflammation that results from it are well described up to date; however, the better understanding of the mechanisms underlying the innate immune system and how to harness them for improving clinical outcomes are yet to be translated into practice and to producing tailor-made therapeutic modalities to mitigate severe disease.

Most of the existing antiviral drugs target the replication machinery of viruses within the cells, while corticosteroids abrogate inflammation. The S protein is essential for binding virion to host cells via ACE2, and a direct action against this protein using monoclonal antibodies is one of the alternative treatment strategies to prevent viral penetration (9). However, these interventions are not so efficacious in the later stages of the disease, in which viral genomic copies are no longer detected in patients. In this stage of the disease, moderate-to-critically ill patients may benefit from immunomodulatory interventions, other than corticosteroids, to tone down the abnormal SARS-CoV-2-mediated inflammatory response.

In this respect, BromAc^®^ is a selective combination of bromelain and acetylcysteine, capable of not only destroying the peptides and disulfide bonds of glycoprotein, including the S protein (10), but also of modulating cytokine storm in tracheal aspirate samples from critically ill COVID-19 patients, and it is an effective mucolytic (11). These attributes make this combination a putative approach for taming SARS-CoV-2-mediated inflammation and tissue damage.

Bromelain is a proteolytic enzyme “soup” obtained from the pineapple plant *Ananas comosus* extract. In addition to its proteolytic activity, this enzyme allows the hydrolysis of glycosidic interactions in carbohydrates found in mucus (12). In parallel, acetylcysteine is an agent used to treat paracetamol overdose (13, 14), a mucolytic agent, and a powerful antioxidant that can reduce disulfide bonds of proteins (15). The combination of these two compounds (BromAc^®^) exhibits a synergistic mucolytic effect *in vitro* and *ex vivo* and clinically for cancer (16) and COVID-19 (11). In this study, we sought to understand the effect of BromAc^®^ in the SARS-CoV-2-mediated cytokine storm *in vitro* and its impact on cellular mechanisms upon *in vitro* stimulation with SARS-CoV-2.

## 2 Materials and methods

**Figure 1:**
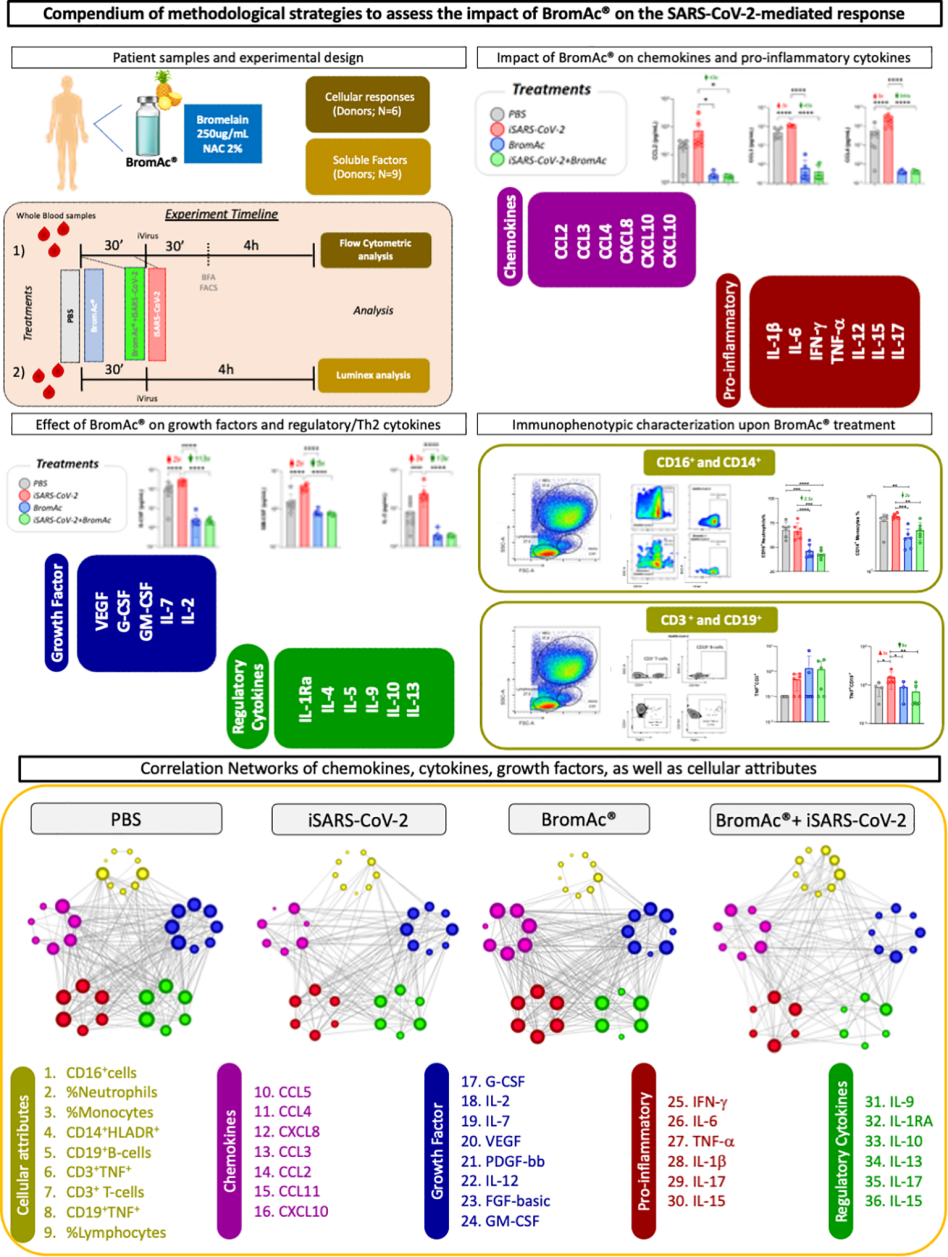
Study design for assessing the anti-inflammatory activity of BromAc^®^ in peripheral blood cells stimulated with inactivated SARS-CoV-2 (iSARS-CoV-2) *in vitro*. Timeline of experimental setup with sequential incubation of BromAc^®^ and iSARS-CoV-2. Samples from healthy donors were employed for the study of soluble mediators (N=9) and for cellular responses (N=6) using immunophenotyping leukocyte analysis by flow cytometry after short-term culture with iSARS-CoV-2 alone in the presence of BromAc^®^. Control cultures (PBS) without iSARS-CoV-2 were tested in the absence and presence of BromAc^®^. Analysis of chemokines (CCL2, CCL3, CCL4, CXCL8, CXCL10, CCL11) and pro-inflammatory cytokines (IL-1β, IL-6, IFN-γ, TNF-α, IL-12, IL-15, IL-17), regulatory cytokines (IL-1Ra, IL-4, IL-5, IL-9, IL-10, IL-13), and growth factors (FGF-basic, PDGF, VEGF, G-CSF, GM-CSF, IL-7, IL-2) was performed using Luminex magnetic bead assay. Immunophenotypic analysis of neutrophils, monocytes, and lymphocytes was performed based on flow cytometric analysis. Systems biology approaches based on network analysis were performed in order to investigate the integrative relationship of soluble mediators and cellular attributes and their connectivity.

**Figure 1** shows the study design and methodological strategies used in the present study.

### 2.1 BromAc^®^

For the preparations, 100 mg of sterile lyophilized bromelain (Mucpharm Pty Ltd) was resuspended in phosphate-buffered saline (PBS), and aliquots were stored at -20°C. Acetylcysteine (NAC) (Link pharma) at 200mg/mL, in injectable form, was kept at room temperature until use. The BromAc*^®^* final formulation was composed of a final concentration of NAC at 2% and bromelain at 250μg/mL.

### 2.2 Patient samples

For the short-term culture assays, a total of nine blood samples from healthy donors (n=9) were used; the demographic information from the volunteers is available in **Supplementary Table 1**. The samples were collected in 9mL sodium heparin tubes and EDTA tubes (BD Pharmingen), and the assays were performed with fresh blood. For the Luminex analysis, the nine samples were assayed. For the Flow cytometric immunophenotypic analysis, 6 samples were evaluated. This study followed the principles of the Declaration of Helsinki and the Ministry of Health resolution No. 466/2012 for research involving human subjects, and it was approved by the institution’s ethics committee (CAAE number: 45919121.6.0000.5526).

### 2.3 Short-term culture of peripheral blood cells

Initially, hemograms were performed with samples collected separately in tubes containing EDTA anticoagulant to obtain the global leukocyte count. The complete blood count tables can be found in **Supplementary Tables 2**, **3**. After calculations were performed, the necessary adjustments to obtain cell preparations with 1x10^7^ leukocytes/mL of the blood collected in heparin tubes were made. Then, the blood samples collected in tubes containing sodium heparin were transferred to 50mL polypropylene conical tubes and centrifuged at 660 x g for 10 minutes at room temperature. After centrifugation, the plasma was removed, an equivalent amount of RPMI 1640 medium (GIBCO - Grand Island, NY) was added, and the cell suspension was homogenized and centrifuged at 660x g for 10 minutes at room temperature. This procedure was repeated, and the cell concentration was adjusted to 1x10^7^ WBC/mL.

After adjustment, the cell suspension was transferred to 14 mL polypropylene tubes (Falcon, Becton Dickinson - BD, USA) and incubated for 30 minutes under constant agitation in an incubator at 37°C and 5% CO_2_ in the absence and presence of BromAc^®^ (250 μg/mL of bromelain and NAC at 2%). BromAc^®^ was added 30 minutes prior to viral stimulation to allow full reconstitution and dilution of the compound to the whole-blood culture, acclimating the system to the active ingredients. After this first acclimation, stimulation with inactivated SARS-CoV-2 - iSARS-CoV-2 at a multiplicity of infection (MOI) of 0.1 - was added to the cultures, followed by incubation under the same conditions mentioned above for 30 minutes. Moreover, control cultures (PBS) were performed in the absence of the virus and control with only BromAc^®^ (250 μg/mL of bromelain and NAC at 2%) without virus. As a functional control (positive control) for the study of cell activation and investigation of intracellular cytokines, cultures were performed in the presence of non-specific stimulation by PMA (phorbol 12-myristate 13-acetate, Sigma Aldrich, USA) and ionomycin (Sigma Aldrich, USA) (data not shown). Subsequently, for studying intracellular cytokine staining, brefeldin A (BFA) (Sigma-Aldrich, USA) was added to all tubes at the final concentration of 10 μg/mL, and the cultures were incubated for 4 hours under the same conditions. Finally, the final concentration of 2mM EDTA was added, and the cultures were incubated for 15 minutes at room temperature and away from light. For studying soluble factors, incubation with different treatments was performed in the absence of brefeldin A. After incubation, the tubes were centrifuged at 660 x g for 10 minutes at room temperature to remove the supernatant for the Luminex analysis.

### 2.4 Assessment of culture supernatant soluble mediator with Luminex^®^

We sought to verify the effect of BromAc^®^ on inflammatory mediators present in the culture supernatant obtained from the *in vitro* system with the peripheral blood samples from healthy patients who were treated with 250 µg of bromelain and 2% NAC (BromAc^®^). The samples from the short-term culture of peripheral blood cells, performed as described above, were first cleaned by centrifugation at 800 x g for 10 min at room temperature, and the supernatants were transferred to fresh 2 mL microtubes. The samples were diluted 1:10 and incubated with magnetic beads coated with monoclonal antibodies specific to various immune mediators: chemokines (CCL2, CCL3, CCL4, CXCL8, CXCL10, CCL11) and pro-inflammatory cytokines (IL-1β, IL-6, IFN-γ, TNF-α, IL-12, IL-15, IL-17), growth factors (FGF-basic, PDGF, VEGF, G-CSF, GM-CSF, IL-7 and IL-2), and regulatory cytokines (IL-1Ra, IL-4, IL-5, IL-9, IL-10, IL-13). The experiments were performed according to the manufacturer’s instructions using the Bio-Plex Pro Human Cytokine 27-plex Assay (Bio-Rad, CA, USA). Immune mediators were measured in culture supernatant samples, and the concentrations of each sample were determined according to standard curves run for each molecule tested, using the analysis based on the fifth-parameter logistic fit curve. The data results were displayed as pg/mL for all soluble mediators in the culture supernatants from the different treatments tested.

### 2.5 Immunophenotyping of the peripheral blood cells

After incubation, the tubes were centrifuged at 660 x g for 10 minutes at room temperature to remove the supernatant for the Luminex test. For assessing viability, an aliquot of cells incubated with the treatments tested including BromAc was stained with Acqua505 viability stain (Invitrogen), followed by flow cytometric analysis, which is shown in **Supplementary Figure 1**. After homogenization, 300µL of the cultures were transferred to 5mL polystyrene tubes (Falcon, Becton Dickinson - BD, USA) containing antibodies for the evaluation of lymphocyte, macrophage, and neutrophil subpopulations. For surface molecule labeling, the antibodies anti-CD3-PE-Cy™7 (Clone SK7), anti-CD16-PE (3G8), anti-CD14-eF450 (61D3), anti-HLA-DR-FITC (G46-6), anti-CD19-PerCP-Cy™5.5 (HIB19), and anti-TNF-α-APC (6401.1111) were employed. IgG1 APC isotype control (clone MOPC-21 – Sigma-Aldrich) was used as a separate tube.

Following a 20-minute incubation period at room temperature and protected from light, red cell lysis was performed by adding 3mL of commercial lysis solution (FACS Lysing Solution - BD, USA) under vortex agitation, and the tubes were incubated for another 10 minutes under the same conditions. Then, the tubes were centrifuged at 600 x g at 18°C for 7 minutes. The supernatant was discarded, and the cell pellet was resuspended and vortexed with 500μL of PBS-W (PBS solution plus 0.5% bovine serum albumin and 0.1% sodium azide). Subsequently, the cells were permeabilized by adding 3mL of PBS-P (PBS-W plus 0.5% saponin), and the tubes were incubated for 10 minutes at room temperature and away from light, followed by centrifugation at 600 x g at 18°C for 7 minutes. After discarding the supernatant, further washing was performed by adding 3mL of PBS-W, and further centrifugation was carried out. The cell pellet was resuspended in 200μL of PBS-W, and the cells were stained with anti-human TNF-α monoclonal antibody, previously diluted in PBS-P.

After a 30-minute incubation at room temperature and away from light, 100 μL of PBS-P was added to each well, and the plate was subjected to centrifugation at 600 xg, 18°C for 10 minutes. The plate supernatant was discarded, and 200 μL of PBS-W was added to each well. After centrifugation at 600 xg and room temperature for 10 minutes, the supernatants were discarded, and the cells from each well were resuspended in 200μL of PBS. Then, 15 minutes post-incubation at 4°C, the analysis of the peripheral blood cell population’s morphometric, phenotypic, and functional parameters was conducted using the LSRFortessa flow cytometer (BD) and analyses performed with FlowJo software (Treestar).

### 2.6 Statistical analysis

The statistical analyses were performed with the GraphPad Prism software, version 8.00 (GraphPad Software, USA). Initially, the normal distribution of the data was considered as parametric or non-parametric using the Shapiro–Wilk test. Then, the Kruskal–Wallis test was applied for non-parametric data and comparison between three or more groups, followed by Dunn’s multiple comparisons test. The Mann–Whitney test was employed for comparative analysis between two independent groups. One-way ANOVA and then Dunnett’s *post hoc* multiple comparisons test between groups were performed for parametric distribution. Statistically significant differences were considered when p<0.05.

Correlation matrices were constructed using correlation analysis employed for assembling integrative networks. Significant correlations calculated using Pearson and Spearman tests at p<0.05 were selected for construction of the networks. The “r” scores were used to build the nets using the Cytoscape software platform (available at https://cytoscape.org) based on circular eccentric layouts. Each node represents a soluble mediator or a cellular attribute. Connecting edges feature strong correlations (“r” scores above |0.67|) amongst pairs of factors. The size of the nodes indicates the number of correlations.

## 3 Results

### 3.1 Evaluation of the immune soluble mediator profile induced by iSARS-CoV-2 on peripheral blood leukocytes *in vitro* after treatment with BromAc^®^

An assay employing peripheral blood leukocyte cells from healthy donors was developed to evaluate the impact of BromAc^®^ on the cytokine storm induced exclusively and directly by the iSARS-CoV-2 virus. The culture supernatant was collected, and the evaluation of soluble mediators, including chemokines, cytokines, and growth factors, was conducted.

**Figure 2:**
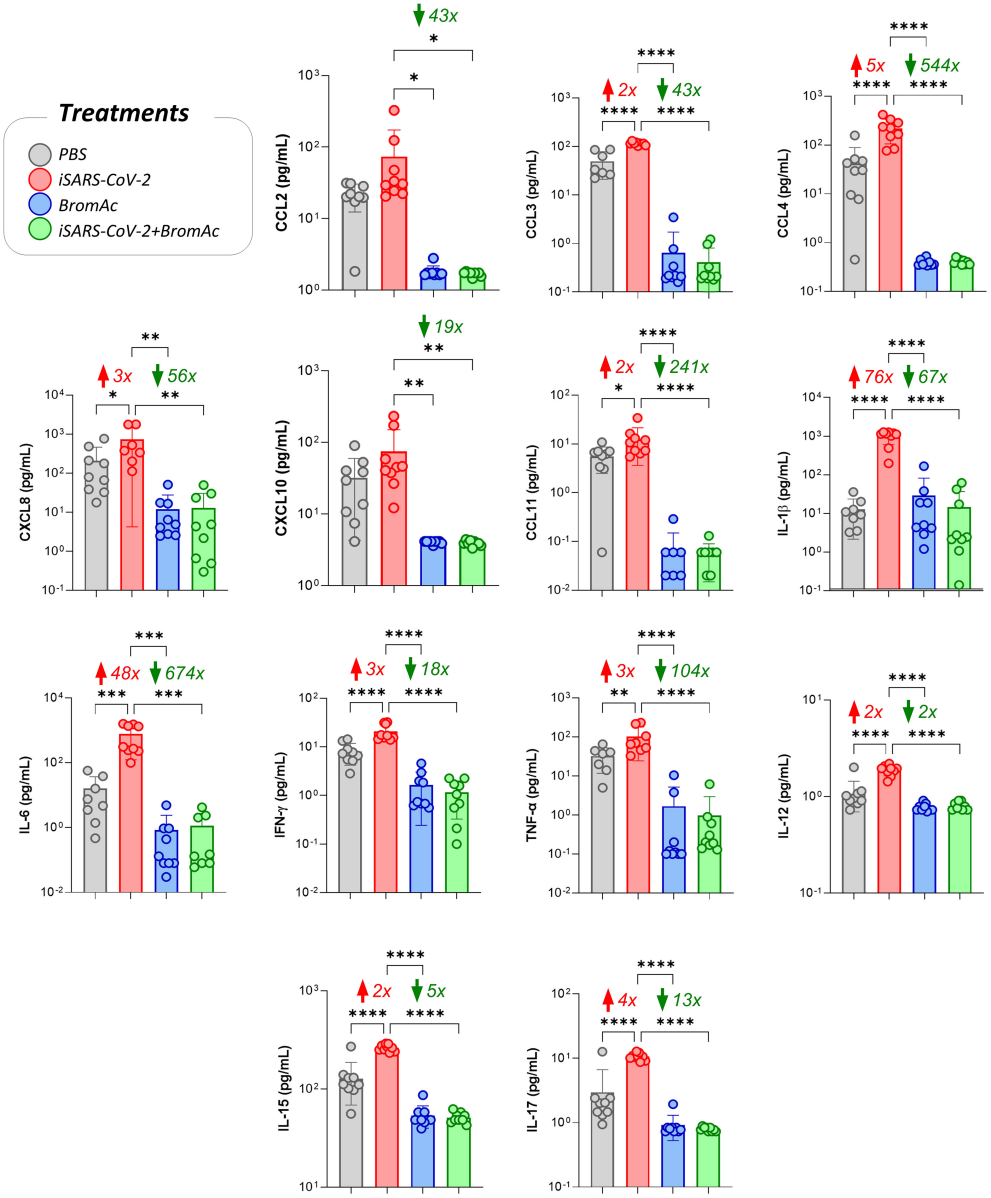
Impact of BromAc^®^ on chemokines and pro-inflammatory cytokines induced directly by iSARS-CoV-2. The soluble mediators were measured by a bead-based multiplex assay as described in the Material and Methods section. Peripheral blood of nine healthy donors was used to evaluate the chemokines (CCL2, CCL3, CCL4, CXCL8, CXCL10, CCL11) and pro-inflammatory cytokines (IL-1β, IL-6, IFN-γ, TNF-α, IL-12, IL-15, IL-17), quantified on culture supernatants obtained from an *in vitro* peripheral blood culture stimulation system with iSARS-CoV-2. The results are expressed in pg/mL and presented as scattering distribution of individual values over bar plots, underscoring the median value on each bar. Significant differences at p<0.05 are indicated by asterisk (*) and connecting lines to the iSARS-CoV-2 culture with the PBS, BromAc^®^, and BromAc^®^ with iSARS-CoV-2 cultures. The red and green arrows indicate the fold-change increase induced by the stimuli and the reductions associated with the treatment, respectively.

The results indicate that SARS-CoV-2, in its inactivated form, was able to induce a robust production of pro-inflammatory cytokines such as IL-1β, IL-6, TNF-α, and IFN-γ in peripheral blood leukocytes from healthy individuals after short-term stimulation. In addition, treatment with BromAc^®^ was able to significantly reduce the secretion of these pro-inflammatory mediators (**Figure 2**).

**Figure 3:**
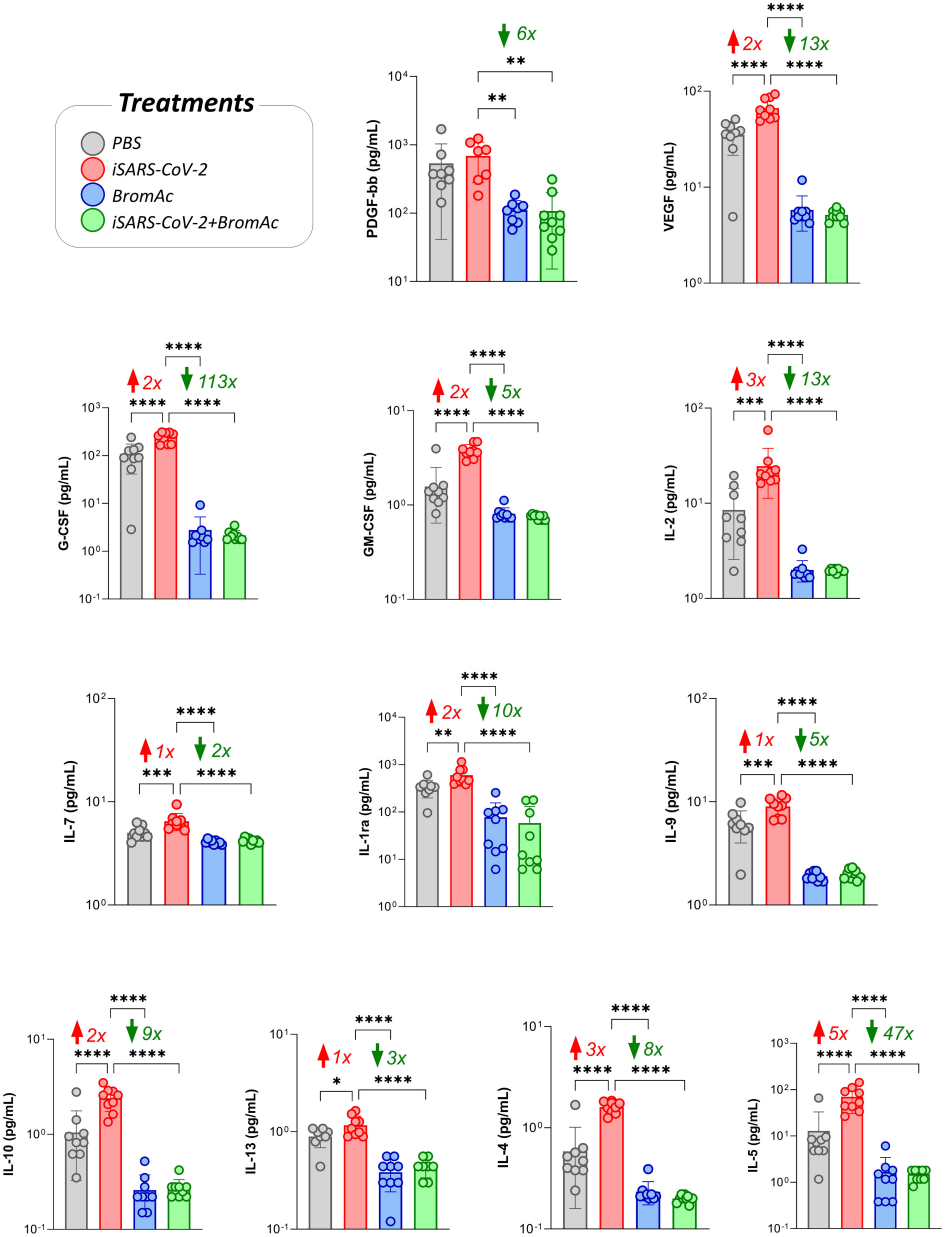
Effect of BromAc^®^ on growth factors and regulatory/Th2 cytokines induced directly by the iSARS-CoV-2 virus. The profile of soluble immunological mediators was evaluated by the bead-based multiplex assay as described in the Material and Methods section. Peripheral blood of nine healthy donors was used to evaluate the growth factors (FGF-basic, PDGF, VEGF, G-CSF, GM-CSF, IL-7, IL-2) and regulatory cytokines (IL-1Ra, IL-4, IL-5, IL-9, IL-10, IL-13), quantified from samples of culture supernatants obtained from an *in vitro* peripheral blood cells stimulation system with iSARS-CoV-2. The results are expressed in pg/mL and presented as scattering distribution of individual values over bar plots, underscoring the median value on each bar. Significant differences at p<0.05 (*), p<0.005 (**), p<0.0005 (***) and p<0.0001 (****) are indicated by asterisk and connecting lines to the iSARS-CoV-2 culture with the PBS, BromAc^®^, and BromAc^®^ + iSARS-CoV-2 cultures. The red and green arrows indicate the fold-change increase induced by the stimuli and the reductions associated with the treatment, respectively.

The analysis of growth factors PDGF-bb, G-CSF, GM-CSF, VEGF, IL-2, and IL-7 indicated that there was an increase in growth factors in the samples stimulated with iSARS-CoV-2 and a reduction in the samples treated with BromAc^®^, as seen in **Figure 3**. Although no increase in platelet recruitment-associated growth factor PDGF-bb was observed upon iSARS-CoV-2 stimuli, reduced steady-state levels of this growth factor were observed upon treatment with BromAc^®^.

Regarding the regulatory cytokines of the Th2 axis, IL-4, IL-5, IL-1Ra, IL-9, IL-10, and IL-13 in the group stimulated with the virus, there was a significant reduction induced by BromAc^®^ (**Figure 3**). These results indicate that BromAc^®^ may contribute to regulating excessive proinflammatory responses, such as type 1 immune responses, against SARS-CoV-2 during the viral phase.

### 3.2 Evaluation of the cellular immune response profile modulated by BromAc^®^ against *in vitro* stimulation with iSARS-CoV-2

To determine the anti-inflammatory profile of BromAc^®^, the immunophenotypic profile of peripheral blood leukocytes was evaluated upon iSARS-CoV-2 stimuli and after treatment with BromAc^®^, as previously described (17). High viability of peripheral blood leukocytes was observed upon treatment with BromAc^®^ (**Supplementary Figure 1**).

**Figure 4:**
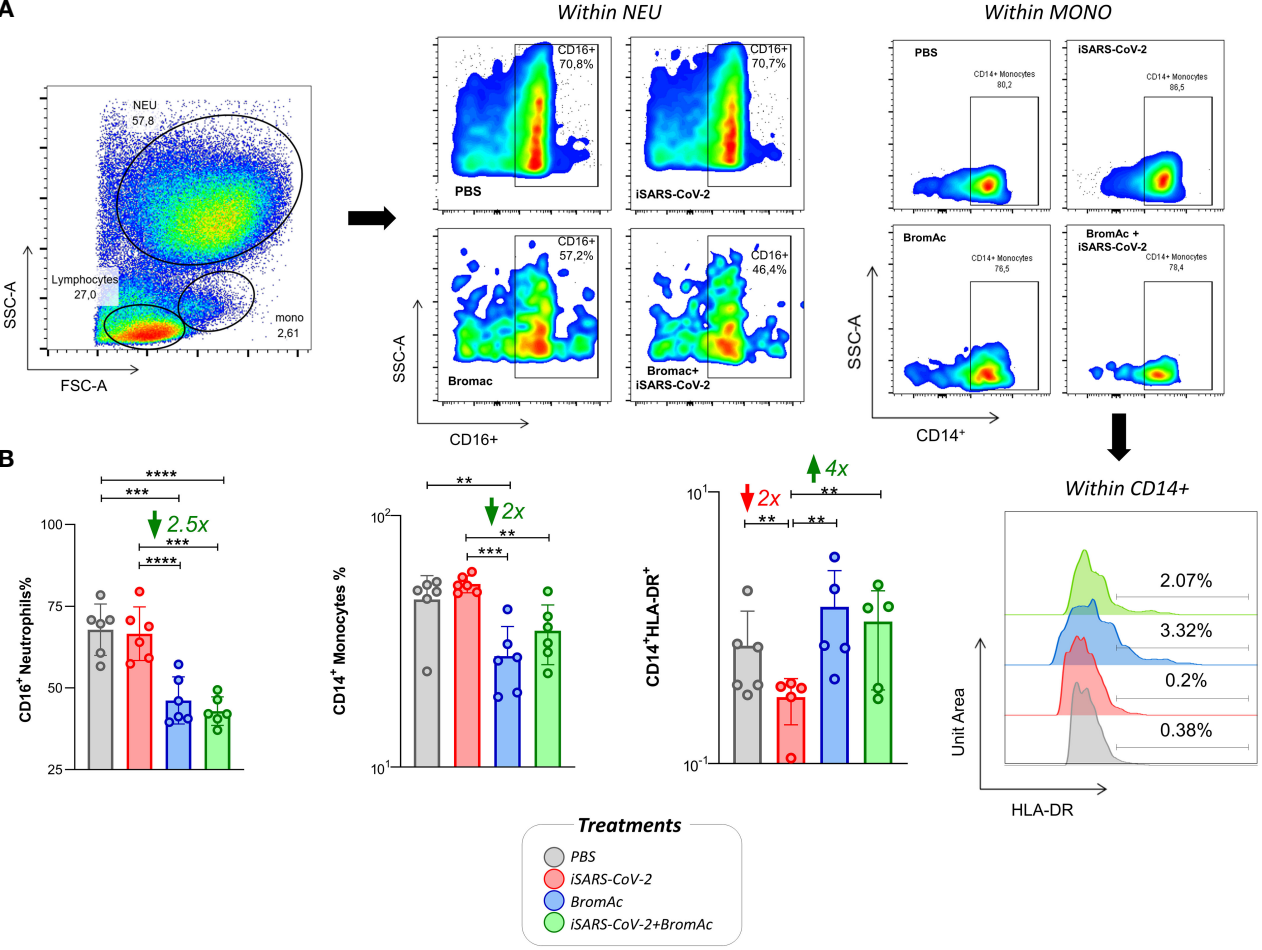
Immunophenotypic characterization and visualization of neutrophil and monocyte subpopulations after iSARS-CoV-2 short-term stimulation and upon BromAc^®^ treatment. **(A)** Size (FSC) vs complexity (SSC) dot plots of a representative healthy donor followed by the CD16 and CD14 marker expression for the different treatments; **(B)** the immunomodulatory profile of BromAc^®^ on neutrophils and monocytes stimulated with iSARS-CoV-2 in an *in vitro* system employing peripheral blood cells obtained from heparinized blood collected from six healthy volunteers. The legend shows the different treatments performed and their respective color key. The results are expressed in % and presented as scattering distribution of individual values over bar plots, underscoring the median value on each bar. Significant differences at p<0.005 (**), p<0.0005 (***) and p<0.0001 (****) are indicated by asterisk and connecting lines to the iSARS-CoV-2 culture with the PBS, BromAc, and BromAc^®^ + iSARS-CoV-2 cultures. The red and green arrows indicate the fold-change increase induced by the stimuli and the reductions associated with the treatment, respectively.

The results show that BromAc^®^ modulated the populations of CD16^+^ neutrophils and CD14^+^ monocytes after stimulation with iSARS-CoV-2. A lower percentage could be observed in cells treated with BromAc^®^ in steady state and in the presence of iSARS-CoV-2 (**Figure 4**). BromAc^®^ treatment was shown to increase the activation marker HLA-DR in CD14^+^ monocyte populations compared to viral stimulation. These results indicate that BromAc^®^ may have a modulatory effect, reducing neutrophil and monocyte populations but, at the same time, preserving and improving the activation of monocytic cells by upregulating HLA-DR during either steady state or upon iSARS-CoV-2 stimuli.

**Figure 5:**
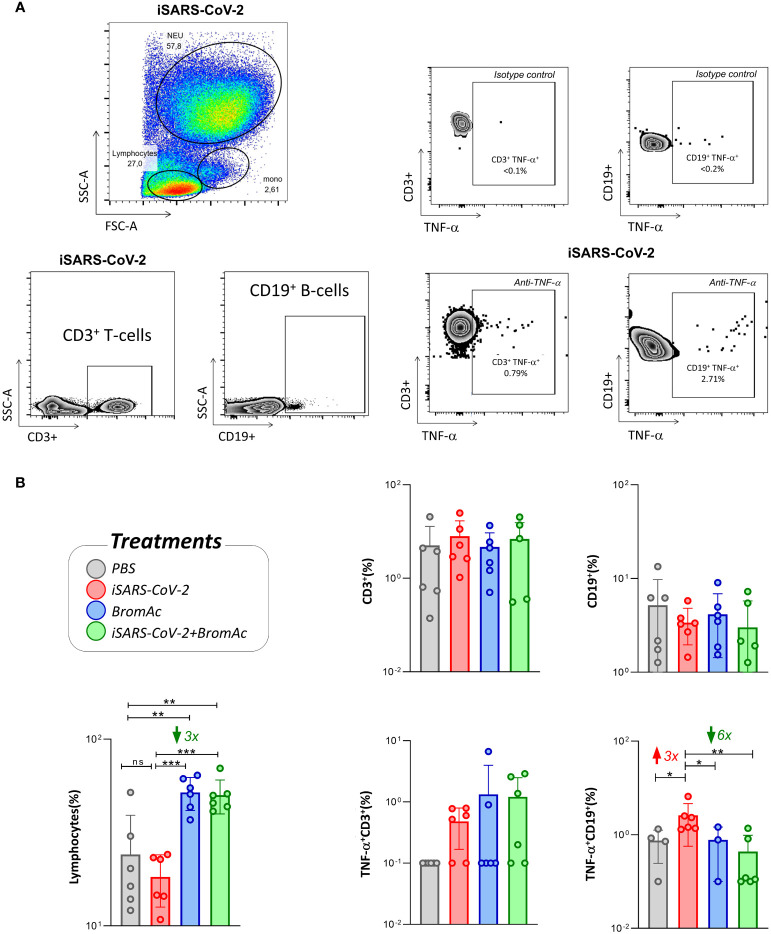
Cellular immunophenotyping for characterization and visualization of lymphocyte subsets and TNF-α production. **(A)** FSC vs SSC dot plots of a representative healthy donor followed by the CD3 and CD19 expression for the iSARS-CoV-2 stimuli, along with the TNF-α cytokine production and isotype controls; **(B)** the immunomodulatory profile of BromAc^®^ on lymphocytes stimulated with iSARS-CoV-2 in an *in vitro* system employing peripheral blood cells obtained from heparinized blood collected from six healthy volunteers. The legend shows the different treatments performed and their respective colors. The results are expressed in % and presented as scattering distribution of individual values over bar plots, underscoring the median value on each bar. Significant differences at p<0.005 (**), p<0.0005 (***) are indicated by asterisk and connecting lines to the iSARS-CoV-2 culture with the PBS, BromAc, and BromAc^®^ + iSARS-CoV-2 cultures. The red and green arrows indicate the fold-change increase induced by the stimuli and the reductions associated with the treatment, respectively.

When evaluating the effect of BromAc^®^ on lymphocyte subpopulations, we observed a significant increase in the lymphocyte levels, regardless of stimulation with iSARS-CoV-2 (**Figure 5**). We also observed that treatment with BromAc^®^ decreased the production of TNF-α by CD19^+^ B cells compared to the group stimulated with the inactivated virus.

### 3.3 Integrative correlation matrices and network relationships between soluble immune mediators and the cellular immune response in culture supernatants stimulated with i SARS-CoV-2

**Figure 6:**
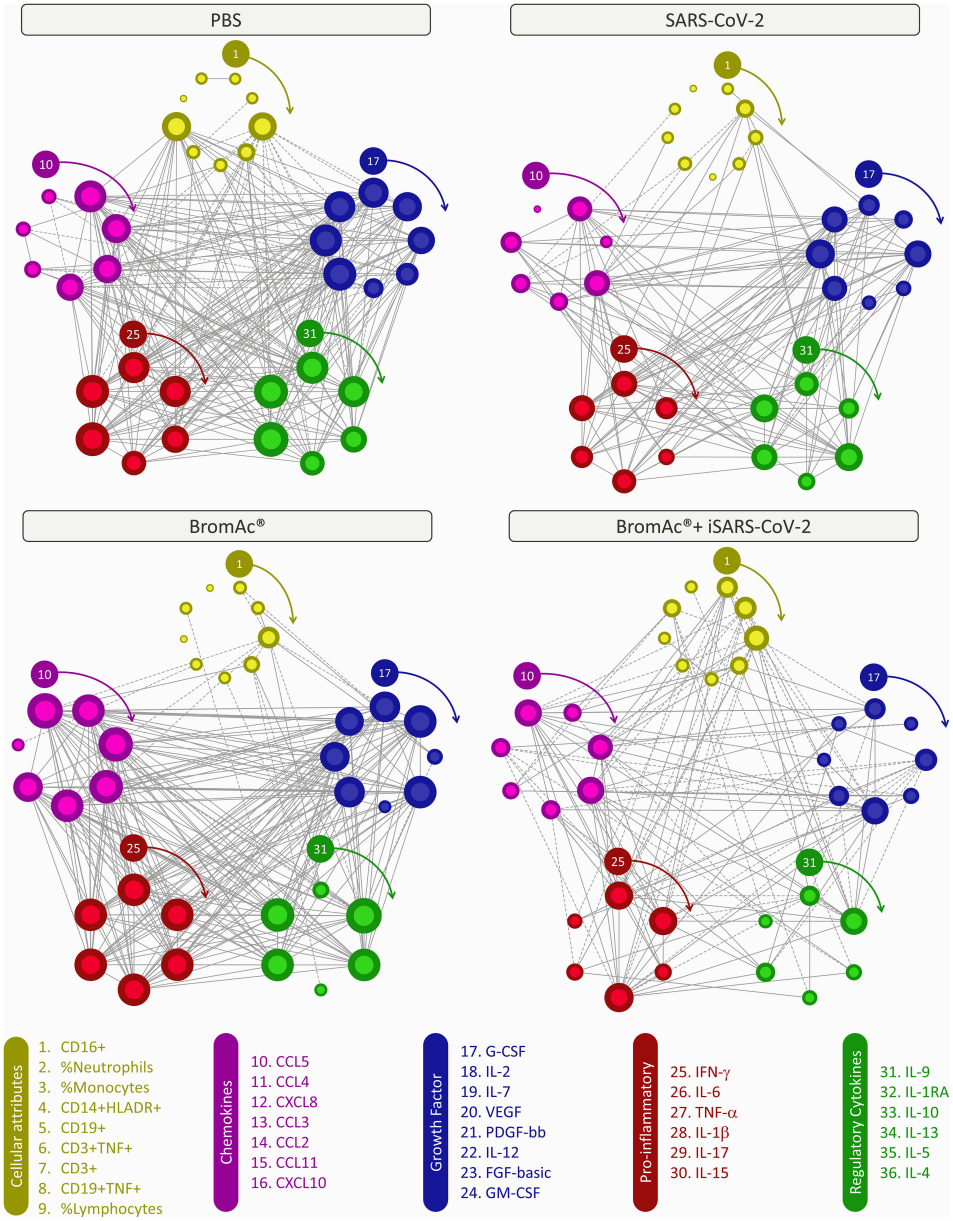
Integrative correlation networks of serum soluble mediators and cellular attributes of an *in vitro* peripheral blood culture stimulation system with iSARS-CoV-2. Comprehensive correlation matrices were assembled based on the Pearson and Spearman “r” scores between chemokines (purple), pro-inflammatory cytokines (red), regulatory cytokines (green), growth factors (blue), and cellular attributes (yellow) measured from an *in vitro* system employing peripheral blood cells obtained from heparinized blood collected from the healthy volunteers. The number of each parameter is shown in the legend above from 1 to 36, and clockwise arrows indicate each of their positions. Networks were built using a circular layout based on eccentricity, considering all significant correlations. Connecting edges illustrate strong correlation (“r” scores ≥ |0.67|, thick gray lines) between pairs of attributes. Negative correlations are underscored by dashed lines. The size of the node indicates the number of connections; the more connections, the larger the node.

Systems biology approaches, utilizing comprehensive correlation matrices and networks, were employed to assess the interaction among soluble mediators and the cellular attributes upon iSARS-CoV-2 stimulation (**Figure 6**). The results show a distinct network for each treatment used, indicating similar networks between PBS and BromAc^®^, with many correlations, while the viral stimulus and BromAc^®^ + iSARS-CoV-2 show a decrease in network connectivity. When evaluating the correlation of the cellular attributes, a major difference could be seen in the correlation lines between all treatments.

Moreover, it was possible to identify selective edges associated with distinct degrees of iSARS-CoV-2, and BromAc^®^. ISARS-CoV-2-stimulated cultures showed pro-inflammatory activity, characterized by the strong protagonism of IFN-γ and IL-15. The connections amongst these pro-inflammatory cytokines are stronger with the growth factors (IL-12, FGF-basic, GM-CSF) and less with the cellular attributes. The high correlation presented in the PBS and BromAc^®^ cultures indicates that the immune response is well regulated since cellular parameters and regulatory cytokines correlate positively between proinflammatory cytokines and chemokines. Conversely, connectivity patterns associated with distinct iSARS-CoV-2 exposure profiles were observed, as demonstrated by the mirrored inverted INF-γ axis, displaying positive correlation (↗) in BromAc^®^ and negative correlation (↘) in BromAc^®^ + iSARS-CoV-2. Importantly, in the BromAc^®^ + iSARS-CoV-2 group, the predominance of moderate connectivity profile as opposed to iSARS-CoV-2 alone may illustrate the disruption of the immunological mechanisms underlying the elevated pro-inflammatory state observed upon viral stimuli without BromAc^®^. Finally, the negative correlations of the cellular subsets in the BromAc^®^ + iSARS-CoV-2 indicate a conspicuous immunomodulatory activity of BromAc^®^, which is featured by the higher negative correlation amongst neutrophils and monocytes with the chemokines (CCL5, CCL3, and CXCL8) and pro-inflammatory cytokines (IFN-γ, IL-6, and IL-1β).

## 4 Discussion

In the present study, we sought to evaluate the impact of BromAc^®^ against iSARS-CoV-2-mediated proinflammatory responses *in vitro*. BromAc^®^ has demonstrated anti-inflammatory activity, reducing the cytokine storm and improving viscosity in the human specimens of tracheal aspirate samples from patients with severe COVID-19. These results with a wider panel of immune soluble factors corroborated previous findings from our group that show robust proteolytic activity in a smaller panel of chemokines and cytokines (11). We have previously shown the synergic anti-inflammatory effect of acetylcysteine together with bromelain, but not alone, in samples from COVID patients (11). In this present study, our focus was to understand the potential of BromAc^®^ in its final formulation in inducing modulatory properties in human peripheral blood cells. Therefore, as it is not an additive effect, the compounds taken separately were not employed in this study design, given that they alone would not account for the synergistic anti-inflammatory effect observed with the final formulation.

The activity of BromAc^®^ was observed on regulatory cytokines such as IL-10. IL-10 has been shown to be augmented in tracheal aspirate samples from critically ill COVID patients that progress to death (18). Not only proinflammatory but also increased regulatory factors need to be addressed during a modulatory response, considering that IL-10 and other regulatory cytokines may contribute to impaired resolutive mechanisms during tissue repair and convalescence associated with long COVID (19, 20).

BromAc^®^ decreased the number of mature SSC^high^FSC^high^CD16^+^ neutrophils post-stimulation with iSARS-CoV-2 on the peripheral blood of healthy patients, possibly by CD16 cleavage, considering that viability was very high after BromAc^®^ treatment. Preliminary results on a phase 1 clinical trial based on the safety of nebulized BromAc^®^ already reveal that this combination is safe for human use in its nebulized form. Neutrophils are one of the most important agents of hyperinflammation that leads to acute respiratory distress in COVID-19. Previous evidence has suggested that neutrophils are directly involved in cytokine secretion, virus internalization, and the production of extracellular neutrophil traps (NETs) during viral infection (6). Furthermore, the presence of neutrophilia is not only systemic in critically ill COVID-19 patients but has been found as increased neutrophil influx locally in lung tissue at autopsy (21). In agreement with our results, previous reports showed that bromelain may reduce the migration of neutrophils into the inflammatory site, and while this may be related to the reduction of cytokines, it is also likely due to the removal of cell surface receptors on neutrophils, including CD44, CD62, and chemokine receptors, and it has effects similar action in tumor cells (22–24).

The same effect of BromAc^®^ is noted on SSC^mid^FSC^high^CD14^+^ monocytes, which, like neutrophils, are recruited to the inflammatory milieu and may contribute to tissue damage. Monocytes can be divided into three classes based on the expression of CD14 and CD16 markers: classical (CD14^+^CD16^-^), non-classical (CD14^dim^CD16^+^), and intermediate (CD14^+^CD16^+^). Under pathological conditions, including viral infections, classical monocytes are mostly activated by inflammatory mediators and by viral infection, thereby infiltrating the affected tissues and acquiring proinflammatory features such as alveolar resident macrophages. In COVID-19, these monocytes can alter glucose metabolism, promoting SARS-CoV-2 replication (25). Although during the infection SARS-CoV-2 may favor the proinflammatory profile of monocytes, our *in vitro* results of iSARS-CoV-2 stimulation promoted decreased expression of HLA-DR, an MHC-II expressed in antigen-presenting cells. In agreement with our findings, an analysis of scRNA sequencing of PBMCs from patients with severe COVID-19 revealed that eight genes encoding HLA class II molecules were downregulated in all study patients, even more significant in ventilator-dependent and elderly patients (26). This mechanism is also observed for other viruses in which they sabotage immune cell function and antigen presentation. BromAc^®^ reversed the HLA-DR downmodulation mediated by iSARS-CoV-2 and promoted a significant increase in its expression on the surface of monocytes. HLA-DR corresponds to an activation and cell function marker for antigen-presenting cells, and improving its expression may render antigen presentation more efficient. The hypothesis by which this increase in HLA-DR is happening may be associated with the reshaping of antigen-presenting cells by BromAc^®^. More specifically, it could be speculated that the cysteine protease complex, from which bromelain can form, is cleaving key proteolytic regulators of Class II MHC processing (27) and, therefore, increasing the initial half-life of nascent HLA-DR in the cell surface of monocytes. It is important to consider that this could be a transient early effect of BromAc^®^ in HLA-DR dynamics, which could change at later time points.

Antigen presentation is a key component that connects convalescence and the development of resolutive processes. They are also known to train T- and B-cell-mediated responses. Although lymphocytes play an essential role in COVID-19 convalescence, excessive cytokine production may promote tissue damage, especially through innate-like B and T cells (28–30). While BromAc^®^ does not seem to impact the T-cell compartment, B cell secretion, more specifically the iSARS-CoV-2-induced TNF-α, by mature CD19^+^ B cells was down-modulated by BromAc^®^. In COVID-19, extreme lymphopenia and alterations in the function of blood B cells and T cells might bring about failure in immunological tolerance, promoting self-reactive antibodies and lasting autoimmune diseases (31). This autoimmune state is portrayed by the clonal expansion of B cells with proinflammatory profile and the production of autoantibodies that may contribute to COVID-19 pathogenesis and pathology. Therefore, B cells may be an important source of cytokines, especially TNF-α, during autoimmune processes such as the ones described for COVID and the long COVID spectrum, while this same cytokine produced by T cells may exert pro-apoptotic and effector functions in killing virus-infected cells. Clinical investigations have suggested that autoreactive immune processes, including rheumatoid arthritis, can develop soon after SARS-CoV-2 infection, featuring one of the outcomes of the long COVID spectrum (32). In agreement with our findings, previous reports suggest that SARS-CoV-2 viral epitopes may activate autoreactive B cells that are associated with cytokine storm and other mediators such as TNF-α, triggering autoreactive responses (33).

In severe cases, cytokine storms are due to the overproduction of inflammatory cytokines, including IL-1β, IL-6, IL-12, IFN-γ, and TNF-α (34). Increased serum levels of IL-7, IL-10, macrophage colony-stimulating factor (CSF) (M-CSF), granulocyte-CSF (G-CSF), granulocyte–macrophage (GM-CSF), interferon gamma-induced protein 10KD (IP-10), monocyte chemoattractant protein (MCP-1), macrophage inflammatory protein (MIP-1), and TNF-α are seen, especially in patients with severe disease (35, 36). There is a strong link between viral infection, inflammation, and mucus production. Several signaling pathways are intertwined by the activity of TNF-α, IL-6, and L-10, which may contribute to triggering sudden mucus hypersecretion along with other mediators of the COVID-19 cytokine storm (37, 38). While viral infection can directly cause the overproduction of sputum by respiratory cells, cytokine storm causes the overproduction of mucus via the STAT, MAPK, and NF-kB pathways in cells and IFN-AhR signaling pathways in COVID-19. Mucus production in respiratory diseases is normally related to inflammation, and in COVID-19, mucin is known to be induced by interferon present in the bronchoalveolar lavage fluid (BALF) of COVID-19 patients and animals (38, 39). Neutrophil and macrophage recruitments may contribute to the composition of a thick and rich mucus production (38). Therefore, it is essential that muco-active therapies may take into consideration the cellular components of mucus for a future immunomodulatory therapy for COVID-19 and other mucus-productive respiratory diseases. We have previously reported on the strong mucolytic ability of BromAc^®^ (11). In addition, cytokines are composed of amino acid sequences that may be substrates for the productive proteolytic cleavage mediated by bromelain. However, there is an additional complexity in that bromelain can stimulate or inhibit cytokine release from cells in different settings, which corroborates our present results (40, 41). Bromelain has been reported to reduce G-CSF, GMCSF, IFN-γ, MIP, and TNF-α by inflamed tissue in intestinal bowel disorders (42, 43).

BromAc^®^ seems to act by proteolytic cleavage of immune soluble mediators induced by the virus. BromAc^®^ may disrupt peptides, including disulfide and O-glycosidic bonds, in protein receptors on the cell surface, but its proteolytic efficiency seems to be higher on the cleavage of soluble factors stimulated by the virus, as can be observed in **Supplementary Figure 2**. BromAc^®^ alone exerts a modulatory effect even at steady state without the presence of viral stimuli. It is well known that even *in vitro* short-term culture conditions bring stress to cells, and the steady state of control cultures reflects a limited but significant level of activation of leukocytes, which was modulated here by BromAc^®^.

Even though the profiles of BromAc^®^ alone at steady state and after viral stimulation by inactivated SARS-CoV-2 present a similar pattern, we believe that the effect of BromAc^®^ at steady state and upon viral stimulation may rely on different and additive synergic mechanisms, considering that the magnitude of response triggered by the virus and the signaling pathways activated by the virus in its inactivated form are divergent from what is activated at steady state. Of note, BromAc^®^ decreased the expression of chemokines CCL2 and CCL3, which are associated with pro-inflammatory macrophage recruitment to the lungs. Decreased levels of pro-inflammatory cytokines IL-1β, IL-6, and VEGF were observed after treatment with BromAc^®^, indicating possible modulation of the inflammasome-associated pathway, which plays a role during COVID-19 (11, 44).

Our integrative analysis using networks of correlations showed that neutrophils gained positive correlations with IL-1β and IL-17 after iSARS-CoV-2 stimulation. Studies indicate that the potential activation of the NLRP3 inflammasome by SARS-CoV-2 directly leads to elevated levels of pro-inflammatory cytokines IL-1β and IL-18 in severe COVID-19 patients, which are associated with adverse outcomes (44). IL-1β is also associated with increased production of TNF-α, and the two cytokines stimulate the production of IL-6, which is also a potent inducer of C-reactive protein (CRP). CRP is a strong and early inflammatory marker, which is elevated in the serum of COVID-19 patients and a predictor of poor outcome (45). Regarding IL-17, this proinflammatory cytokine plays a role in the development of the acute respiratory distress syndrome (SARS) by enhancing neutrophil infiltration into the lungs. IL-17 signaling amplifies the pathological inflammation process that leads to cytokine release, particularly IL-6 and IL-1β (46). Conversely, there is an increase in negative correlations in the cellular cluster, especially concerning the connections of cells with pro-inflammatory cytokines such as IFN-γ, IL-1β, and IL-6 after BromAc^®^+iSARS-CoV-2 treatment. These results may suggest that cytokines loose synchrony with cells after treatment with BromAc^®^ during viral stimuli, which corroborates the anti-inflammatory effect of BromAc upon SARS-CoV-2-mediated cytokine storm.

This study has some limitations. The fact that the *in vitro* system used in the present study does not fully replicate the *in vivo* COVID syndrome in humans is expected. It is important to bring about the limitations of the *in vitro* cell culture models in replicating the full spectrum of immune responses observed in COVID-19 patients. The immune response in COVID-19 patients is influenced by various factors, including host-specific factors, viral load, viral variants, and the overall systemic environment (34). These multifactorial elements cannot be fully recapitulated in cell culture conditions. Additionally, cell culture systems often lack the interplay of immune cells with other tissues and organs and the influence of the host’s overall health status. Emerging research suggests that the interactions amongst SARS-CoV-2, lymphocytes, and neutrophils are more complex than previously thought (47, 48). It is also important to highlight that we used the inactivated virus, which lacks the ability to infect these and other cultured cells. Therefore, it is expected to find divergencies in the *in vitro* system as compared to the *in vivo* COVID-19 disease in humans. However, we were able to recapitulate cytokine storms and cellular activation during iSARS-CoV-2 stimuli, which were clearly abrogated by BromAc^®^. These results indicate that, although limited, the *in vitro* model was an appropriate system to test the ability of BromAc^®^ to tame the cytokine storm and cellular responses induced by the virus.

## 5 Conclusion

The results of this study indicate a robust anti-inflammatory effect of BromAc^®^ in taming SARS-CoV-2-specific cytokine storm, indicating its potential as a therapeutic for COVID-19 and other pro-inflammatory respiratory conditions. Further assessment of the mucolytic and anti-inflammatory effects of BromAc^®^ will be examined in a planned upcoming randomized phase 2 study.

## Data availability statement

The original contributions presented in the study are included in the article/**Supplementary Material**, further inquiries can be directed to the corresponding authors.

## Ethics statement

The studies involving humans were approved by Ethics Committee of Universidade Estadual de Santa Cruz (UESC) and Federal University of Minas Gerais (UFMG). The studies were conducted in accordance with the local legislation and institutional requirements. The participants provided their written informed consent to participate in this study.

## Author contributions

GF: Conceptualization, Data curation, Formal Analysis, Investigation, Methodology, Writing – original draft, Writing – review & editing. FC: Data curation, Formal Analysis, Writing – review & editing. ÁR: Data curation, Formal Analysis, Writing – review & editing, Methodology. LG-d-P: Data curation, Formal Analysis, Investigation, Writing – review & editing. LC: Conceptualization, Supervision, Validation, Writing – review & editing. OF: Data curation, Funding acquisition, Writing – review & editing. FF: Funding acquisition, Resources, Writing – review & editing. MT: Funding acquisition, Resources, Writing – review & editing. AS: Funding acquisition, Resources, Writing – review & editing. ME: Resources, Writing – review & editing, Conceptualization. DM: Funding acquisition, Supervision, Writing – review & editing, Resources. SV: Conceptualization, Funding acquisition, Supervision, Writing – review & editing. JC-d-R: Conceptualization, Formal Analysis, Funding acquisition, Methodology, Project administration, Supervision, Writing – original draft, Writing – review & editing.
