# Supplementary material for: Taming the SARS-CoV-2-mediated proinflammatory response with BromAc®
Source: Front Immunol. 2023 Dec 13;14:1308477. doi: 10.3389/fimmu.2023.1308477 (PMC10773902; doi:10.3389/fimmu.2023.1308477)

**
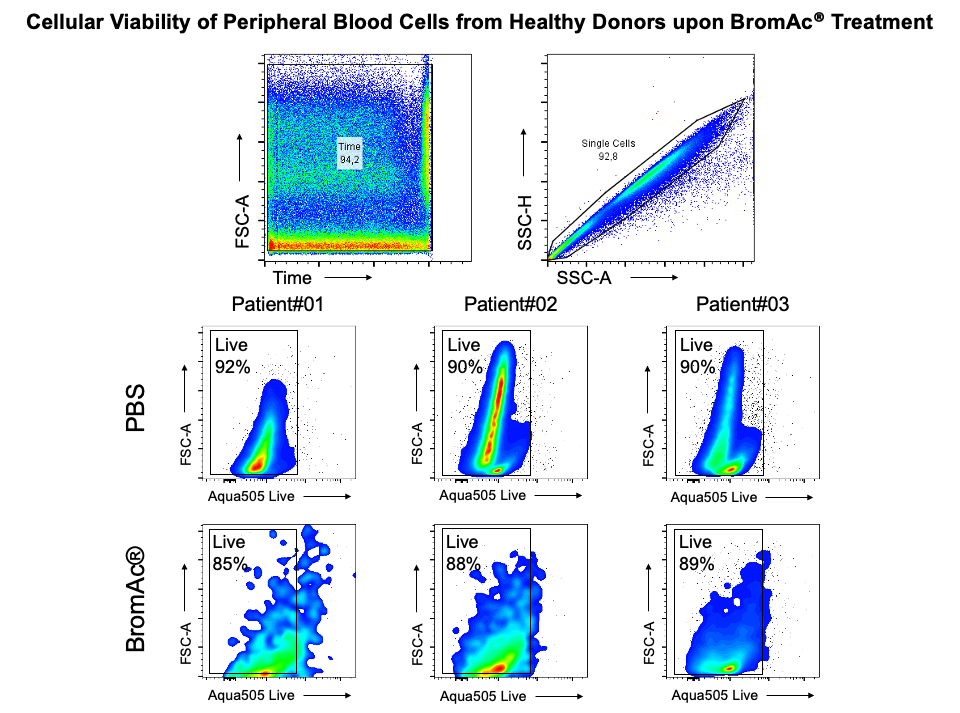
**

**Supplementary Figure 1 - Cellular viability analysis of blood peripheral cells upon treatment with BromAc^®^ assessed by Flow Cytometry.** Cell viability was measured after incubating cells in the presence of BromAc^®^ up to 48 hours. Staining with viability dye Acqua505 was performed after incubation and data acquisition was carried out by flow cytometry. Graphs displaying FSC vs time, followed by FSC vs SSC pseudocolor plots were prepared to select single cells. Within single cells, viability was assessed by drawing FSC vs Acqua505 fluorescence channel. Live cells were selected amongst the unstained our low intensity quadrant. Graphs are representative of 3 healthy donor samples. Percentages of live cells are expressed for PBS-treated controls as well as BromAc^®^-treated samples.

**
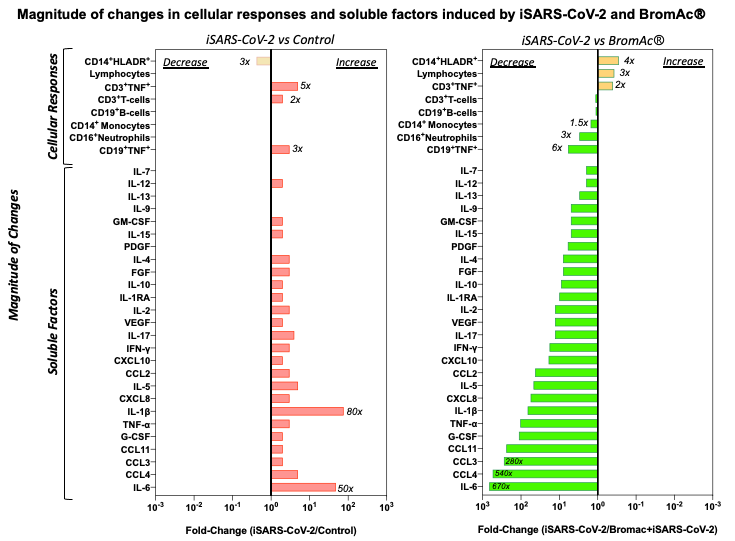
**

**Supplementary Figure 2 - Magnitude of changes in cellular responses and soluble immune factors from healthy donors upon iSARS-CoV-2 stimuli and treatment with BromAc^®^.** The magnitude of changes in the cellular responses tested, the levels of chemokines (CXCL8, CCL11, CCL3, CCL4, CCL2, CXCL10), pro-inflammatory cytokines (IL-1β, IL-6, TNF-α, IL-12, IFN-γ, IL-15, IL-17), regulatory cytokines (IL-1Ra, IL-4, IL-5, IL-9, IL-10, IL-13) and growth factors (FGF-basic, PDGF, VEGF, G-CSF, GM-CSF, IL-7 and IL-2) were calculated as the mean fold-change ratio of tubes stimulated with iSARS-CoV-2 over the PBS control (left panel, red bars) or the mean fold-change ratio of iSARS-CoV-2 over the tubes treated with BromAc^®^ in addition to iSARS-CoV-2 stimulation (right panel, green bars). Measurements of soluble mediators were carried by Luminex Bio-plex platform as described in material and methods section. The results are expressed as mean values and presented in bar chart format as ascendant fold change magnitude for BromAc^®^ treatment. The panel highlights the fold-change with decreased or increased levels which were underscored in the graphs.


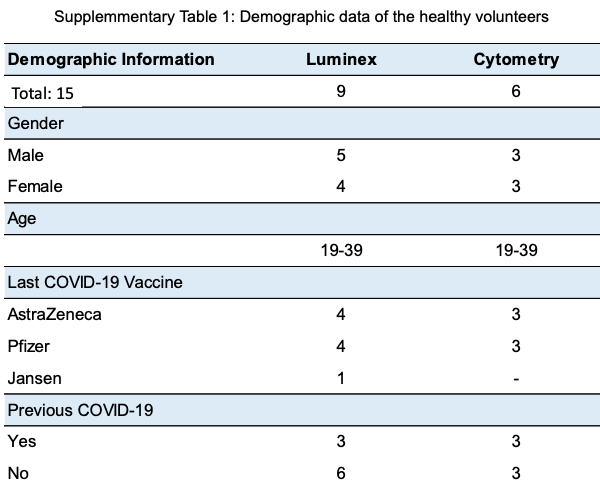

Supplement: Supplementary file 1 [file DataSheet_1.docx]
